# Reflections on the Constraints and Opportunities in Therapy in Rett Syndrome

**DOI:** 10.1100/tsw.2006.186

**Published:** 2006-08-25

**Authors:** Alison M. Kerr

**Affiliations:** Division of Community Based Sciences Academic Centre Gartnavel Royal Hospital, Glasgow G12 0XH, United Kingdom

**Keywords:** Rett syndrome, United Kingdom, United Kingdom

## Abstract

More than 20 years of clinical and research experience with affected people in the British Isles has provided insight into particular challenges for therapists, educators, or parents wishing to facilitate learning and to support the development of skills in people with Rett syndrome. This paper considers the challenges in two groups: those due to constraints imposed by the disabilities associated with the disorder and those stemming from the opportunities, often masked by the disorder, allowing the development of skills that depend on less-affected areas of the brain. Because the disorder interferes with the synaptic links between neurones, the functions of the brain that are most dependent on complex neural networks are the most profoundly affected. These functions include speech, memory, learning, generation of ideas, and the planning of fine movements, especially those of the hands. In contrast, spontaneous emotional and hormonal responses appear relatively intact. Whereas failure to appreciate the physical limitations of the disease leads to frustration for therapist and client alike, a clear understanding of the better-preserved areas of competence offers avenues for real progress in learning, the building of satisfying relationships, and achievement of a quality of life.

## INTRODUCTION

The Rett disorder is due to mutation of the gene *MECP2* at the tip of the X chromosome[1,2] and is responsible for at least 80–90% of cases presenting with "classic" Rett syndrome[3]. The classic picture was first described in young girls, when the condition was thought to be degenerative and confined to females[4,5,6]. The occurrence of a developmental regression at around 1 year of age led to the initial assumption that the child was born healthy and then suffered inevitable deterioration, but it has since become clear that the disease is not progressive[7]. It is recognised in many adults and in a few boys[8], and it is evident that although the earliest signs are subtle and frequently missed, the disease affects the development and function of the brain even before birth[9,10]. It is also appreciated that there is a very wide range in severity[11]; the most severe and the mildest cases, as might be expected, showing the cardinal signs to a greater or lesser degree than the "classic" case[11]. This variation in severity is due in part to the fact that all female cells have two X chromosomes, but use only one. The variable extent to which the neurones are dependent on the X chromosome with the faulty gene, therefore, influences the severity of the condition in that person. The exact position of the mutation in the gene *MECP2* and other factors determining the expression of the gene also affect severity. Experience in the U.K. suggests that a remarkably consistent group of clinical problems is to be found in affected cases, however mild or severe they may be. It is the constraints and opportunities associated with this highly characteristic Rett disorder profile that will be explored here.

## METHODS

The development of Rett syndrome support organisations in 1985 in Scotland (RSA Scotland) and England (RSAUK) made possible a working relationship with the author through which the organisations funded a medical advisory service for families and their professional advisers. This included teaching sessions and clinics at which people with Rett could be examined at the request of their families. The wealth of resulting clinical experience laid the foundation for further clinical, epidemiological, and neurophysiological research and a nation-wide survey recording the health of each reported person, i.e., the British Isles Survey (BIS or BIRS), based until 2005 in Glasgow, now transferred to the University of Wales Institute of Medical Genetics in Cardiff. Reliable data from all sources are entered into a database[12]. Key items are recorded at 5-year intervals from birth and throughout life. A simple BIS scoring system for severity has been validated against later severity and survival[13]. The score includes muscle tone disturbance, locomotion, scoliosis, occurrence of epilepsy, and feeding difficulty. The early score has been shown to rise until 15–20 years of age and then to stabilise[13]. A separate BIS health score is based on the items: persistent epilepsy, episodes of respiratory or other illness, body weight, and the parent's opinion of health[14]. It is through these activities that reports and observations have been gathered, indicating practical problems and successes in therapy.

### Why is the Clinical Profile of the Rett Disorder so Specific?

In Rett disorder, the fault in the gene *MECP2* (note italic notation) leads to failure of production of its active protein MECP2 (nonitalic notation) that is important for the normal development and function of the brain[15,16]. It also influences the expression of several other genes that are important for brain growth and function. *MECP2* normally plays a part in the brain stem regulation of heart rate and breathing, and is of great importance in the cerebral cortex[17]. As the brain grows and matures throughout infancy and childhood, the affected neurones fail to make adequate connections with each other. That failure profoundly disturbs the functions that are most dependent on multiple neuronal connections, the “neural networks”. Most severely affected, therefore, are finely controlled movement, speech, thought, memory, and imagination, while many functions that rely less on complex neuronal interactions are relatively spared. This pervasive, but selective, pattern of abnormality in the brain leads to the distinct pattern of disability, the “disease profile”, that is characteristic for the disorder. It is important to note that this profile is remarkably consistent regardless of the level of severity[18]. For example, hand use and speech are always impoverished, even in the least-affected people who may speak and write[18]. Posture and balance are always measurably disturbed, even in those who can walk and run. Peripheral reception of sight and sound appears normal, while central processing of these signals is defective and this is true of both severe and mild cases[19]. Control of heart rate, blood pressure, and respiratory rhythm appears to be disturbed in everyone, although varying in intensity[20].

## CONSTRAINTS AND HIDDEN OPPORTUNITIES

While progress in research provides the hope that it will become possible to prevent or rectify the defects in the brain, much can now be achieved in reduction of the impact of these on function and quality of life for the person with Rett and the family.

## The Primary Need for Assessment

As for any person with disabilities, accurate diagnosis and a thorough functional assessment are the foundation for therapy, and such assessments should be repeated at intervals throughout life to ensure that the individual's needs are met and possibilities recognised[21]. Such assessments benefit from the expertise of a range of therapists. In the U.K., we call on the services of the physiotherapist, occupational therapist, music therapist and other communication therapists, dietician, optometrist, and audiometrician. When such expertise is not available, the careful observations of the family or caregiver may still provide a valuable guide to care and provision. Families and caregivers are quick to learn from the professionals, and their observations should always be recorded as part of a formal assessment. The aim of the assessment is to discover and minimise the impact of disabilities, and to discover and support competences.

## Posture and Locomotion

Posture is invariably disturbed in Rett. There appears to be reduced appreciation of the position of the body in space (proprioception). There is also disturbance of the tension in muscles (muscle tone) required to support the body, and to allow smooth and accurate movement. That area of function is dependent on continuous monitoring and adjustment by the complex neural networks in the brain that are specifically disabled in Rett. In the youngest children, the defect is manifest as reduced muscle tension (hypotonia), “floppiness”, most marked in the most severe. This reduced muscle tone is usually superseded by an abnormal increase in tone (hypertonia or dystonia), usually first evident at the ankles (gastrocnemius muscle), finally affecting most areas. The most severely affected children cannot stand or walk without support, and in everyone there is a tendency for contractures to develop at the joints. Acute dystonic episodes are common and involuntary movements may affect muscles in the face, limbs, and spine. The increased muscle tension commonly affects some muscle groups more than others and one side of the body more than the other. In the spine, this probably contributes to the almost invariable development of scoliosis, requiring surgical correction in about half[22]. Early detection of these evolving problems gives the best opportunity for the provision of appropriate support. The therapist must understand and accommodate these disabilities in any plans.

At least as important for the individual is encouragement and the opportunity to be active, using the whole body as fully as possible. It is in such activity that the hidden movement skills of people with Rett usually become apparent. The families’ observations are of particular value. It will be seen that although planned movements are poor, spontaneous movements and postural adjustments can be surprisingly fluent and appropriate in such activities as managing a swing, swimming, horse riding, and dancing. Such skills clearly give satisfaction to the individual and may improve with practice, benefiting body and mind. Triggers for such useful spontaneous actions appear to include a strong level of motivation combined with low demand and a relaxed environment. Since music appeals to almost everyone with Rett, it has become one of the most highly valued of the therapies and may be combined with other physical therapies to relax and to motivate the individual, as well as holding a major place in communication therapy[23]. Water at a comfortable temperature in a pleasant, supported environment is also an excellent facilitator for many people with Rett and physiotherapy may be most successful in such a pool.

## Involuntary Movements

The extraordinary stereotyped movements and the failure of useful hand activity characteristic in Rett reflect the inability of the brain to organise finely controlled hand movements and the release of more primitive alternating patterns[24]. Research studies indicate that the individual has very little control of these repetitive rhythms. The movements increase with agitation and abate with relaxation, and so may mislead the therapist into believing that they constitute a form of intentional communication. On the contrary, useful communication is sometimes assisted when these movements are interrupted. As with the unexpected postural and locomotor skills, the best use of the hands tends to be seen when the individual is well motivated, not stressed by demands, and allowed plenty of time to act in a relaxed atmosphere[25].

## Feeding and Nutrition

Feeding difficulties are common in all but the mildest cases. These include positioning difficulties, inadequate mouth closure, interruption of feeding by involuntary movements, erratic respiratory rhythm, inability to chew and move food around the mouth, aspiration of food or fluids into the lungs, reflux of acid material from the stomach leading to reflux oesophagitis, and ingestion of air with sometimes painful bloating[26]. When severe, these difficulties necessitate specialist assessment and in some cases direct percutaneous nutrition. A good nutritional state is essential for everyone and without it existing skills are likely to be lost and other problems exacerbated. Although expert support may be necessary, it should also be kept in mind that simple strategies (such as withdrawal from a noisy dining room with extra time for assistance) may reduce the most severe difficulties at meal times.

## Control of Cardiac and Respiratory Rhythm

The involuntary abnormal breathing patterns in Rett also require understanding and accommodation by the therapist. Breathing becomes irregular when even the least-affected people become agitated, and a variety of abnormal rhythms are commonly present whenever the individual is awake and alert. These may include breath holding, deep breathing, and inadequate ventilation[27]. These disturbances may cause further agitation so that a vicious circle is produced. Severely disturbed respiratory rhythm stresses the already defective control of blood pressure and heart rate leading to several types of nonepileptic "vacant spells"[27,28]. Interestingly, such attacks very rarely culminate in an epileptic seizure. True epilepsy, associated with disturbance of the electrical discharges in the cortex, also occurs in about half of all cases and should be treated with anticonvulsant medication when severe. However, nonepileptic vacant spells are much more common than epilepsy and major difficulties arises when these are mistakenly medicated as intractable epilepsy. When in doubt, brain stem and electroencephalographic monitoring are recommended[27]. Any such interruption of awareness seriously interferes with performance and the therapist has to make allowance for such events.

## Mood

Sudden mood swings are especially common in children during the early infancy regression period, but occur also in older children and adults with Rett disorder. During regression, the levels of the excitatory neurotransmitter glutamate are known to be abnormally high[29] and this may result in agitation and pain. A quiet situation with soft music, favoured by the individual, may allow relaxation and permits the use of skills. Of course, people with Rett are at least as likely as any healthy person to develop painful and irritating conditions, and a careful medical examination is always required to search for such causes of distress. Frustration and anger at unwelcome changes and events are also to be expected in sensitive people who cannot express their feelings, and signs of distress always require careful review of the circumstances.

## Communication

Communication perhaps presents the greatest challenge to the therapist because the expected means of communication are interfered with to such a great extent by the Rett disorder and yet the person with Rett loves to communicate[18]. This means that the usual ways to assess communicative capacities are invalid.

Although speech begins to develop in about half of the Rett population, it usually disappears with the onset of the regression period and rarely reappears when that abates. Even in the least severely affected people who can talk and answer questions, the understanding and use of speech is severely restricted. Such speech as is possible gives the impression that in these relatively mildly affected people, the intellect is also greatly affected by the disease[18].

In marked contrast is the remarkable ability of people with Rett to relate warmly to other people, to enjoy company, to appreciate simple interpersonal jokes, and to enter into the spirit of joint activities. They have clear preferences and can use their own repertoire of actions and expressions very effectively to indicate these[11]. Personality is well preserved throughout life and reactions to situations are often age appropriate. This area of competence provides enormous rewards for therapist and “client”, but demands great patience, sensitivity, and ingenuity to observe and exploit the possibilities. Music has been described as the “genius” for Rett work[11]. Not only has music the potential to provide the relaxed environment that facilitates spontaneous actions, but it offers also the individual at any age rich possibilities for choice and self-expression through active participation[30]. It should be understood, however, that to be of value, music must be used just as carefully as speech. Loud or continuous sound of any kind becomes a source of stress and should be avoided. Since vision and hearing are usually normal in Rett, there should be periodic routine assessments to ensure that any necessary support is provided.

## CONCLUSIONS

Challenges for the therapist have been discussed that may be improved by physical, pharmacological, or surgical treatment, but otherwise must be accommodated by the therapist: reduced use of the hands, stereotyped hand movements, and other unwanted involuntary movements; irregular breathing with episodes of altered awareness; feeding difficulties; epilepsy; periods of agitation; altered sense of body position and, therefore, reduced ability to stand and walk; tendency to develop contractures and scoliosis; the lack of speech; and intellectual restriction.

Coexisting with these severe and complex difficulties and often obscured is the potential for learning and the use of skills, present at all ages, provided that the individual is maintained in good health. The challenge to the therapist is to discover these competences and find means by which they can be enjoyed: the capacity for spontaneous movements (reaching, touching, swimming, swinging, dancing, riding); pleasure in such activities; sociability and the capacity for specific and maintained relationships; sensitivity to musical cadence and rhythm; good vision and hearing with evident enjoyment of visual, tactile, and auditory experiences; retention of the capacity to learn and to acquire new skills throughout life.

## ACKNOWLEDGEMENTS

The author wishes to acknowledge collaboration over many years with the colleagues listed in the references, individuals with Rett Syndrome, and their families. The work has been part-funded from several sources including RSAUK, RSA Scotland, IRSA, RSRF, and Quarrier's Homes. The University of Glasgow Department of Psychological Medicine provided the base for my activities during the last 10 years.
